# Air Pollution and ST-Segment Depression in Elderly Subjects

**DOI:** 10.1289/ehp.7737

**Published:** 2005-03-14

**Authors:** Diane R. Gold, Augusto A. Litonjua, Antonella Zanobetti, Brent A. Coull, Joel Schwartz, Gail MacCallum, Richard L. Verrier, Bruce D. Nearing, Marina J. Canner, Helen Suh, Peter H. Stone

**Affiliations:** ^1^Channing Laboratory, Brigham and Women’s Hospital, Department of Medicine, Harvard Medical School, Boston, Massachusetts, USA; ^2^Environmental Epidemiology Program, Department of Environmental Health, and; ^3^Environmental Statistics Program, Department of Biostatistics, Harvard School of Public Health, Boston, Massachusetts, USA; ^4^Cardiology Division, Brigham and Women’s Hospital, Department of Medicine, Boston, Massachusetts, USA; ^5^Division of Cardiology, Beth Israel Deaconess Medical Center, Department of Medicine, Harvard Medical School, Boston, Massachusetts, USA; ^6^Environmental Science and Engineering Program, Department of Environmental Health, Harvard School of Public Health, Boston, Massachusetts, USA

**Keywords:** air pollution, cardiology, elderly, particles, ST-segment depression, traffic

## Abstract

Increased levels of daily ambient particle pollution have been associated with increased risk of cardiovascular morbidity. Black carbon (BC) is a measure of the traffic-related component of particles. We investigated associations between ambient pollution and ST-segment levels in a repeated-measures study including 269 observations on 24 active Boston residents 61–88 years of age, each observed up to 12 times from June through September 1999. The protocol involved continuous Holter electrocardiogram monitoring including 5 min of rest, 5 min of standing, 5 min of exercise outdoors, 5 min of recovery, and 20 cycles of paced breathing. Pollution-associated ST-depression was estimated for a 10th- to 90th-percentile change in BC. We calculated the average ST-segment level, referenced to the P-R isoelectric values, for each portion of the protocol. The mean BC level in the previous 12 hr, and the BC level 5 hr before testing, predicted ST-segment depression in most portions of the protocol, but the effect was strongest in the postexercise periods. During post-exercise rest, an elevated BC level was associated with −0.1 mm ST-segment depression (*p* = 0.02 for 12-hr mean BC; *p* = 0.001 for 5-hr BC) in continuous models. Elevated BC also predicted increased risk of ST-segment depression ≥0.5 mm among those with at least one episode of that level of ST-segment depression. Carbon monoxide was not a confounder of this association. ST-segment depression, possibly representing myocardial ischemia or inflammation, is associated with increased exposure to particles whose predominant source is traffic.

Numerous studies have demonstrated associations of acute increases in particle levels with increased risk of cardiac morbidity and mortality (Pope et al. 1995). Efforts have been directed toward understanding mechanisms for these associations. Canine studies showing increased risk of myocardial ischemia (Wellenius et al. 2003) and a chamber study showing decreased brachial artery diameter with particle exposure (Brook et al. 2002) have provided supportive evidence for particle-induced ischemia as a potential mechanism. Both carbon monoxide and particle mass < 2.5 μg/m^3^ (PM_2.5_) were associated with increased risk of ST-segment depression during repeated submaximal exercise tests among subjects with coronary heart disease in 45 adults with stable coronary heart disease in Helsinki, Finland (Pekkanen et al. 2002); PM_2.5_ was believed to be the primary source of this association, but because of correlation with CO, the authors reported that independent effects were difficult to separate. Black carbon (BC) may be a more precise measure than PM_2.5_ of the portion of particle mass related to traffic (Laden et al. 2000). We examined whether there were independent associations of the ambient traffic-associated pollutants, BC and CO, with ST-segment depression before and after submaximal exercise in a community-based repeated-measures study of elderly adults from Boston, Massachusetts.

## Materials and Methods

### Study design and protocol.

We recruited a panel of elderly subjects living at or near an apartment complex located within 1 km of a central site monitoring station. A baseline screening questionnaire was administered regarding medications, pulmonary and cardiac symptoms, and smoking history. A resting 12-lead electrocardiogram (ECG) was performed. Exclusion criteria included unstable angina, atrial flutter, atrial fibrillation, or paced rhythm. Each subject was assigned a day of the week and a time of day for weekly testing, with the goal of 12 weekly visits during the summer of 1999. Each week, participants were administered a brief questionnaire regarding chest pain, medication changes, and whether medications had been taken that morning. Continuous Holter monitoring with electrodes in a modified V5 and aVF position was performed using the Marquette Seer Digital Recorder (Marquette Inc., Milwaukee, WI). The protocol (Gold et al. 2000) consisted of *a*) 5 min rest, *b*) 5 min standing, *c*) 5 min exercise outdoors (if the participant felt able, a standard walk was performed, involving one climb up a slight incline), *d*) 4 min supine recovery, or *e*) 3 min 20 sec slow, paced breathing (for each of 20 respiratory cycles, the participant was asked to breathe in for 5 sec and then out for 5 sec, coached by a technician).

### Processing of Holter recordings.

The digital Holter recordings were downloaded to a MARS Ultra 60 playback system (Marquette Inc.) for analysis. ST-segments were evaluated for the average value for each portion of the protocol and for possible ischemia, defined as reversible horizontal or downsloping ST-segment depression ≥0.5 mm, a level associated with adverse cardiac risk in patients with acute coronary syndrome (Cannon et al. 1997). Recordings were visually scanned by an experienced analyst to censor artifacts. Custom algorithms were created to calculate the average “ST-segment level” or value, referenced to the P-R isoelectric values, for each portion of the protocol. Separately, each candidate episode of reversible ST-segment deviation was evaluated as possibly representing ischemia, by using real-time ECG strips examined by an experienced analyst and physician blinded to air pollution status. A table of J-point values, ST-segment values, ST-segment slope, and heart rate was printed for each candidate episode beginning 10 min before each episode and ending 10 min after the resolution of each episode. The ST-segment value 60 msec after the J-point was used to define the ST-segment deviation and the ST-segment slope.

### Air pollution measurements.

Air pollution measurements (PM_2.5_, BC, CO) were collected at a central site within 0.5 km of the residences of the subjects, which were on the same busy street trafficked by diesel-powered buses and trucks as well as cars of commuters. Measurements of sulfur dioxide, ozone, and nitrogen dioxide were obtained from state monitoring sites in Boston. Continuous PM_2.5_ was measured using a tapered element oscillating microbalance (TEOM; model 1400A; Rupprecht and Patashnick, Albany, NY). The TEOM sample filter is heated to 50°C, leading to season-specific temperature-related loss of semivolatile mass. Season-specific calibration factors were used to correct for the losses of mass (Allen et al. 1997). The calibration factors were obtained by regressing continuous PM_2.5_ concentrations averaged over 24-hr periods on the corresponding collocated integrated 24-hr Harvard Impactor (Air Diagnostics Environmental Inc., Harrison, ME, USA) low-volume Teflon filter gravimetric measurements.

In the summer in Boston, BC measurements are surrogates for carbonaceous particles, components of PM_2.5_, many of which derive from traffic (local or transported). BC data from this instrument, using the internal empirically determined conversion factor, have correlated well with elemental carbon (Hansen and Rosen 1984). BC was measured using a model AE-14 aethalometer (Magee Scientific Inc., Berkeley, CA). CO was measured continuously with a gas analyzer (model 48; ThermoEnvironmental, Franklin, MA) using a U.S. Environmental Protection Agency (EPA) reference method (Automated Reference Method: RFCA-0981-054).

### Statistical analyses.

For each portion of the protocol, we analyzed the effect of pollution on between-visit, within-subject changes in mean ST-segment level. A standard model for analyzing repeated measures on the same individual is the linear mixed model, which accounts for residual correlation among observations taken on the same subject by including normally distributed random intercepts and pollutant slopes in a linear regression model. Descriptive statistics for ST-segment values, however, revealed skewness in the subjects’ baseline values, making the normality assumption on the random intercepts untenable. As a result, we used two alternative approaches to analyzing the data from each portion of the protocol. First, treating ST-segment level as a continuous outcome, we used a conditional linear mixed model (Verbeke and Molenberghs 2000), which estimates the within-subject effect of a pollutant after conditioning out each subject’s baseline value. This corresponds to putting subject into the linear model as a fixed effect, while specifying the linear slope of pollutant as a random effect (Verbeke and Molenberghs 2000).

The Exposure and Risk Assessment for Fine and Ultrafine Particles in Ambient Air (ULTRA) study has demonstrated the importance of selecting a vulnerable population when seeking to investigate whether pollution influences ECG changes consistent with ischemia (Pekkanen et al. 2002). Although we did not, as in the ULTRA study, have a cohort selected for coronary artery disease, our aim was to evaluate particle pollution effects on elderly individuals with a tendency to develop ST-segment depression, with some ECG evidence for vulnerability to the outcome of interest. Therefore, *a priori*, for each part of the protocol for analyses treating ST-segment level as a continuous outcome, we included only vulnerable subjects, defined as those whose mean ST-segment values for that part of the protocol were negative at least two times during the study (23 of 28 study participants). Analyses were repeated including all study participants to assess the sensitivity of results to the exclusion criteria and to the presence of outliers.

In addition to analyses evaluating ST-segment level as a continuous outcome, we analyzed the binary response “ST-segment depression ≥0.5 mm,” defined as a mean ST-segment level for a given portion of the protocol of at least −0.5 mm (i.e., mean ST-segment level ≤−0.5 mm compared with ST-segment level > −0.5 mm). This definition differed from that of classic ischemia in that it did not require within-test or within-portion of the protocol reversibility. For this secondary analysis, we fit a logistic regression model with random intercepts to data from those subjects having at least one response of each type (depressed and nondepressed ST-segment) during that particular protocol (13 of 28 study participants contributed data to at least one portion of the protocol).

Twenty-four study participants with 269 observations were included in analyses either with continuous or with binary (dichotomous) ST-segment outcomes. We had sufficient observations to evaluate the effects of between-test increases in pollution levels on between-test depression in the mean ST-level for each portion of the protocol. However, we were unable to assess the effect of between-test changes in pollution on the risk of within-test reversible ST-segment depression that fit criteria for ischemia because of the rarity and lack of variability of such events. During the study, only 5 of 28 study participants had ischemic ECG events (defined above as within-test reversible horizontal or down-sloping ST-segment depression ≥0.5 mm).

Each regression model included an indicator variable for each subject, pollutant concentration, a cubic effect of the mean of the current hour temperature, and a linear trend of time. Other confounders considered included day of week and time of day, which were both highly correlated with the subject indicator variables and were thus dropped from the model. Separate models were fit using lags of 1–24 hr, as well as previous 12 and 24 hr moving averages, of pollution concentration. Finally, models containing multiple pollutant concentration as predictors were fit to account for confounding due to moderate to high correlations among different pollutant concentrations. Multiple lags and moving averages were evaluated to select the best lag structure for temperature and each individual pollutant, and models reflect these evaluations. All statistical analyses were performed using the SAS statistical software package (SAS Institute Inc., Cary, NC). The conditional linear mixed models were fit using PROC MIXED, whereas the logistic mixed models were fit using PROC NLMIXED (SAS Institute Inc.).

Estimates of the effects of BC were scaled to the difference between the 10th and the 90th percentile in levels for the appropriate lag or mean value of BC.

## Results

The median age of the population was 73, and many participants had cardiac risk factors (e.g., history of hypertension, prior smoking) or coronary artery disease (Table 1). As expected, mean heart rate rose during exercise and returned to baseline at rest (Table 2) during the 269 tests for the 24 participants included in analyses. Simultaneously, median ST-segment level was lower during and immediately after exercise than at first rest. ST-segment depression was rare in the modified aVF lead, and all subsequent analyses are based on findings in the modified V5 lead, the lead that most consistently identifies myocardial ischemia when it is present (Lanza et al. 1994). Air pollution levels were only modestly elevated, and maximum levels for U.S. EPA criteria pollutants were all below accepted or proposed National Air Quality Standards (Table 3). CO levels never exceeded 2 ppm. BC levels rose early in the morning and were at their peak between 0600 and 0900 hr.

**Figure 1:**
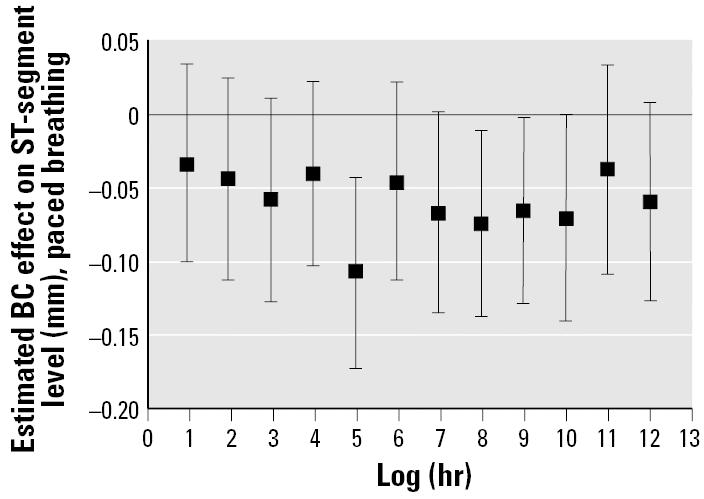
Estimates of the effects of BC on mean ST-segment level during paced breathing, scaled to the difference between the 10th and the 90th percentile in levels for individual hourly lags. Error bars indicate 95% CIs.

Individual hourly lag models showed consistent negative associations of ST-segment level with increased BC for the first 12 hr before testing (Figure 1), but with waning effects after 12 hr. The strongest association between BC and ST-segment level was for the 5-hr lagged value of BC (Table 4). For each portion of the protocol in the continuous models, higher 5-hr BC predicted lower between-test mean ST-segment levels. There was also a consistent effect of the mean of the BC levels during the 12 hr before testing on between-test ST-segment depression. Higher BC levels were also associated with lower between-test ST-segment levels, when averaged (for each individual, for each testing session) over all portions of the protocol (12-hr mean BC: estimated overall ST-segment change = −0.08 mm; *p* = 0.03; 5-hr BC: estimated change = −0.10 mm; *p* = 0.004), suggesting a pollution effect sustained throughout the protocol. Although they were also consistently negative, associations of ST-segment depression with the mean of BC during the 24-hr before testing were weaker, and the BC levels 2 days before testing had no association with ST-segment depression. There was no effect of air pollution on changes in ST-segment level from the rest to exercise or from the exercise to recovery portions of the protocol. The effects of BC on ST-segment depression were not modified by medication use, diagnosis of coronary artery disease, hypertension, sex, or ethnicity.

For the smaller group who had at least 0.5 mm depression at one or more visits, increases in BC were associated with an elevated risk of ST-segment depression ≥0.5 mm, although confidence in the estimates was limited by the smaller numbers of observations (Table 4). The largest estimated risk occurred during the rest period immediately after exercise, when there was a 10.4-fold risk [95% confidence interval (CI), 1.3–83.0] of having between-test ST-segment depression ≥0.5 mm. Although CO was associated with ST-segment depression in single-pollutant models, in multiple-pollution models only BC remained associated with ST-segment depression (Table 5).

## Discussion

In elderly subjects, we found that increases in levels of ambient BC in the 12 hr before testing were associated with between-week depression in the mean ST-segment levels that was present throughout the testing session, with the strongest effects occurring in the postexercise recovery portions of the protocol, a period of cardiac vulnerability in patients with coronary artery disease (Frolkis et al. 2003). There was no effect of pollution on within-testing session changes in the magnitude of ST-segment depression. The risk of ST-segment depression of ≥0.5 mm was elevated with higher pollution; new ECG depression of this magnitude has been associated with increased risk of adverse cardiac events among patients with acute coronary syndrome (Cannon et al. 1997).

Although we found pollution to be associated with ST-segment depression sustained throughout the testing session, the Finnish portion of the ULTRA study found associations of pollution with reversible exercise-induced ST-segment depression (Pekkanen et al. 2002). The etiology of the ST-segment depression we observed is unclear but may represent the consequences of subclinical myocardial ischemia, inflammation, or both.

Although a minority of our subjects had documented coronary disease, many had risk factors predisposing them to subclinical disease and possible ischemia. Particle pollution may decrease myocardial oxygen supply and increase the risk of cardiac ischemia due to epicardial coronary disease through potentially interrelated mechanisms, including systemic inflammation, oxidative stress, endothelial dysfunction, and/or autonomic dysfunction (Gold et al. 2000; Liao et al. 1999). Coronary artery disease is now considered, in large part, an inflammatory process (Ridker et al. 2000), and transient increases in air pollution could lead to transient exacerbation in vascular inflammation. Particle pollution has been linked to ST-segment changes in healthy canines (Godleski et al. 2000) and to reduction of the time to ischemic changes in canines with partial coronary artery occlusion (Wellenius et al. 2003). Brachial artery diameter, which is correlated with coronary artery diameter, was diminished in healthy subjects after exposure in a chamber to concentrated ambient particles (Brook et al. 2002), concomitant with elevated levels of endothelin.

Rather than causing subclinical ischemia, pollution-associated systemic inflammation may lead to low-grade myocardial inflammation, with associated subtle repolarization changes, including sustained ST-segment depression. A series of epidemiologic studies have found associations of particle pollution with elevation of measures of systemic inflammation, including plasma viscosity (Peters et al. 1997), fibrinogen (Gardner et al. 2000), neutrophil count, vascular cellular adhesion molecule and soluble intracellular adhesion molecule (Salvi et al. 1999), and C-reactive protein (Peters et al. 2001).

In this same study, in the entire cohort, we found that BC was associated with a decrease in heart rate variability, suggesting traffic-particle–associated autonomic dysfunction (Schwartz et al. In press). Future work will focus on whether ambient pollution leads to ST-segment depression and autonomic dysregulation through related pathways (e.g., inflammation) or through separate pathways.

BC can be viewed as a surrogate for traffic-related particle pollution; exhaust emissions from diesel-powered vehicles have been identified as the main source of BC or elemental carbon in urban areas (Janssen et al. 2002; Schauer et al. 1996). Laden et al. (2000), in a study of six U.S. cities, found that traffic particles were more strongly associated with cardiovascular deaths than were particles from coal burning. Although BC influenced ST-segment depression, we did not find independent effects of CO on ST-segment level, perhaps because of the low levels of exposure. In one study, short-term exposure to CO, producing carboxyhemoglobin levels of 2–3.9%, were associated with ischemic ST-segment changes in exercising subjects with coronary disease (Allred et al. 1989), although these low-level effects were not reproduced in a study by Sheps et al. (1987). ST-segment depression during exercise was associated with PM_2.5_ and CO in the Finnish study of subjects with stable coronary heart disease who performed repeated biweekly submaximal exercise tests over a 6-month period (Pekkanen et al. 2002). In that study, correlation between the two pollutants made it more difficult to separate their effects. In our Boston setting, CO was not an independent predictor of ST-segment depression. An alternative explanation for the lack of independent associations of the gases with ST-segment depression is more misclassification of exposure, particularly because all the gases other than CO were measured at distances farther than the site where BC and PM_2.5_ were measured, which was very close to the health effects testing site (discussed above).

This study was limited by lack of personal exposure measurements for CO and particles. However, ambient levels were measured on the same busy city street as the participant residences, < 0.5 km away, and studies in Boston have shown that ambient concentrations are good surrogates of personal exposures to PM_2.5_ of ambient origin (Rojas-Bracho et al. 2000). Moreover, the consequence of using ambient particle measures to estimate exposure is likely to be a modest underestimation of pollution effects (Zeger et al. 2000). Our ability to investigate interactions between participant characteristics such as beta-blocker use and particle effects was limited by the size of the population. Confidence in and generaliz-ability of our estimates for our dichotomous outcome were limited by small numbers of observations. Although our cohort was vulnerable on the basis of age, previous smoking, or hypertension history, our potential to document overt ischemic episodes was also limited by the choice of a population, only 18% of whom had diagnosed clinical coronary artery disease. Even in the ULTRA study of a population with doctor-diagnosed coronary artery disease subjected to submaximal exercise, sufficient episodes to examine the outcome of ischemia were documented only among Finnish participants and not among participants from the two other countries (Pekkanen et al. 2002). Our primary analyses did include one individual who, on 3 of 12 visits, reported smoking one to three cigarettes or cigarillos within the previous 48 hr. His data met the inclusion criteria for examining the dichotomous ST-segment depression ≥0.5 mm only during the exercise period; exclusion of this individual from analyses did not influence our findings. In our continuous analyses, we included only those whom we considered vulnerable on the basis of ST-segment depression (23 of 28). A sensitivity analysis showed that although inclusion of the entire cohort somewhat attenuated the magnitude and significance of the results, a significant association of 5-hr BC with ST-segment depression was still detectable during the postexercise period [second rest and paced breathing; e.g., second rest effect estimates: −0.11 vs. −0.08, *p* = 0.001 vs. 0.007, for a subcohort with at least two episodes of ST-segment depression vs. the entire cohort (233 vs. 317 observations)].

In conclusion, in a population of elders susceptible to cardiovascular pollution effects on the basis of age or underlying cardiovascular disease, we found an association between traffic-related particles and ST-segment depression that may represent ischemia or myocardial inflammation.

## Figures and Tables

**Table 1:** Participant characteristics [*n* (%)].

		ST-segment analysis	
Characteristic	Entire cohort (*n* = 28)	Continuous outcome*^a^* (*n* = 23)	Dichotomous outcome*^b^* (*n* = 13)
Sex			
Male	7 (25)	5 (22)	3 (23)
Female	21 (75)	18 (78)	10 (77)
Race/ethnicity			
Black, non-Hispanic	8 (29)	6 (26)	3 (23)
White	19 (68)	16 (70)	9 (69)
Other	1 (4)	1 (4)	1 (8)
Cigarette smoking			
Never	11 (39)	10 (43)	4 (31)
Former	16 (57)	13 (57)	8 (62)
Current	1 (4)	0	1 (8)
Ever asthma*^c^*	1 (4)	1 (4)	1 (8)
Coronary artery disease (ever angina or heart attack)	5 (18)	5 (22)	4 (31)
Ever congestive heart failure	2 (7)	2 (9)	1 (8)
Ever hypertension*^c^*	11 (39)	10 (43)	5 (38)
Medication use			
Beta-blocker	5 (18)	4 (17)	1 (8)
Calcium channel blocker	3 (11)	3 (13)	2 (15)
Angiotensin-converting enzyme inhibitor	7 (25)	7 (30)	4 (31)
Age [median years (range)]	73 (60–89)	71 (61–88)	76 (62–88)

Percentages may not add up to 100 because of rounding.

a Analyses assess the association of pollution with ST-segment level.

b Analyses assess the association of pollution with ST-segment depression ≥0.5 mm.

c Report of doctor’s diagnosis of disease.

**Table 2:** Median*^a^* heart rate and ST-segment level for six protocol periods.

	First rest	Blood pressure	Standing	Exercise	Second rest	Paced breathing
Heart rate (beats/min)	65*^b^*	—	78	86	67	65
ST-segment level, modified V5 lead (mm)	−0.13	−0.10	−0.08	−0.29	−0.27	−0.17
ST-segment level, modified aVF lead (mm)	0.12	0.12	0.11	0.10	0.05	0.10

a Median of the mean values for each part of the protocol, for observations included in analyses. Based on 269 observations on the 24 subjects in analyses using either the continuous or dichotomous outcomes.

b Median heart rate for the period that includes both first rest and blood pressure portions of the protocol.

**Table 3:** Ambient pollution and temperature levels during Holter monitoring (*n* = 269).*^a^*

Pollutant	10th percentile	50th percentile	90th percentile	Maximum
BC (μg/m^3^)				
5-hr*^b^*	0.66	1.28	2.25	4.34
12-hr mean*^c^*	0.79	1.14	1.68	2.23
PM _2.5_ (μg/m^3^)				
5-hr*^b^*	3.8	9.5	25.6	41.0
12-hr mean	4.1	9.8	25.9	35.6
CO (ppm)				
5-hr*^b^*	0.20	0.53	1.08	1.55
12-hr mean	0.38	0.56	0.81	1.04
O_3_ (ppb)				
1-hr	8.5	27.1	54.9	95.4
5-hr*^b^*	2.9	13.3	28.8	57.7
12-hr mean	8.2	19.7	34.2	58.9
NO_2_ (ppb)				
5-hr*^b^*	11.9	22.4	35.6	53.1
12-hr mean	14.3	21.4	35.2	48.9
SO_2_ (ppb)				
5-hr*^b^*	1.3	3.5	8.6	17.4
12-hr mean	2.0	4.3	6.5	11.5
Temperature (°C)	17.2	23.3	28.9	33.3

a Pollutants include daily BC, PM_2.5_, O_3_, NO_2_, SO_2_, and CO. Temperature is current 1-hr mean.

b The distribution of the levels (total *n* = 269) during the fifth hour before Holter monitoring.

c The mean of the levels during the 24 hr before Holter monitoring.

**Table 4:** Ambient BC as a predictor of ST-segment level for five protocol periods.

Outcome variable	No. of observations	5-hr BC*^a^*	*p*-Value	12-hr mean BC*^a^*	*p*-Value
Estimated ST-segment change in mm (95% CI), for continuous outcome*^b^*					
First rest	207	−0.11 (−0.20 to −0.02)	0.02	−0.10 (−0.19 to −0.01)	0.03
Blood pressure	209	−0.09 (−0.16 to −0.01)	0.02	−0.08 (−0.15 to −0.01)	0.03
Standing	196	−0.11 (−0.21 to −0.01)	0.03	−0.09 (−0.19 to 0.01)	0.09
Exercise	257	−0.08 (−0.17 to 0.00)	0.06	−0.02 (−0.11 to 0.06)	0.57
Second rest	233	−0.11 (−0.18 to −0.05)	0.001	−0.07 (−0.14 to −0.01)	0.03
Paced breathing	219	−0.11 (−0.17 to −0.04)	0.001	−0.08 (−0.14 to −0.01)	0.02
Estimated relative risk (95% CI), for ST-segment depression ≥0.5 mm					
First rest	90 (29)*^c^*	5.1 (0.9 to 28.0)	0.06	3.8 (0.7 to 21.3)	0.11
Blood pressure	66 (22)	6.0 (0.8 to 44.8)	0.07	5.7 (0.6 to 56.3)	0.11
Standing	66 (28)	9.2 (1.1 to 78.3)	0.05	8.3 (0.8 to 81.9)	0.06
Exercise	114 (38)	0.9 (0.2 to 4.7)	0.86	0.6 (0.1 to 3.1)	0.53
Second rest	90 (48)	10.4 (1.3 to 83.0)	0.03	2.8 (0.5 to 14.3)	0.19
Paced breathing	66 (22)	6.6 (0.9 to 50.0)	0.06	3.5 (0.5 to 23.6)	0.15

a Estimated for a 10th to 90th percentile change in BC.

b Repeated-measures regression models contain pollution concentration, a cubic effect of current temperature, and a linear trend of time.

c Numbers in parentheses in this column represent the number of positive events with ST-depression ≥0.5 mm.

**Table 5:** 5-hr BC and CO as predictors of continuous ST-segment level in single- and multiple-pollutant models.

Outcome variable, model	Predictor variable	Coefficient	Estimated effect [mm (95% CI)]	*p*-Value
Second rest				
1	BC	−0.07	−0.11 (−0.17 to −0.05)	0.001
2	CO	−0.15	−0.13 (−0.22 to −0.04)	0.007
3	BC	−0.06	−0.09 (−0.17 to 0.03)	0.04
	CO	−0.05	−0.05 (−0.17 to 0.07)	0.45
4	PM_2.5_	−0.0002	−0.004 (−0.08 to 0.07)	0.92
5	O_3_	1.38	0.04 (−0.05 to 0.12)	0.39
6	NO_2_	−1.96	−0.05 (−0.12 to 0.03)	0.22
7	SO_2_	−3.19	−0.02 (−0.10 to 0.05)	0.53
Paced breathing				
1	BC	−0.07	−0.11 (−0.17 to −0.04)	0.001
2	CO	−0.11	−0.09 (−0.19 to 0.00)	0.05
3	BC	−0.07	−0.11 (−0.20 to −0.03)	0.01
	CO	0.01	0.01 (−0.11 to 0.13)	0.87
4	PM_2.5_	−0.0008	−0.02 (−0.09 to 0.05)	0.64
5	O_3_	0.85	0.02 (−0.06 to 0.11)	0.60
6	NO_2_	−1.54	−0.04 (−0.11 to 0.04)	0.33
7	SO_2_	−5.15	−0.04 (−0.11 to 0.03)	0.30

Repeated-measures regression models contain pollution concentration, a cubic effect of current temperature, and a linear trend of time. All models except model 3 include only the single pollutant described. Model 3, for second rest and for paced breathing, includes both BC and CO; thus, the coefficient for BC is adjusted for CO. Results presented are estimated for a 10th to 90th percentile change in BC.
